# PTPN6-EGFR Protein Complex: A Novel Target for Colon Cancer Metastasis

**DOI:** 10.1155/2022/7391069

**Published:** 2022-02-11

**Authors:** Gang Liu, Yan Zhang, Yun Huang, Xinpu Yuan, Zhen Cao, Zhanwei Zhao

**Affiliations:** General Surgery Department, No. 6 Medical Center, General Hospital of Chinese PLA, Beijing 100048, China

## Abstract

This study investigates the expression of nonreceptor protein tyrosine phosphatase 6 (PTPN6) gene in different colon cancer cells and its effect on malignant biological behavior. The expression level of PTPN6 mRNA in different colon cancer cell lines was detected by qPCR. CCK-8, clone formation assay, scratch assay, and transwell assay were used to detect the effect of knockdown or overexpression of the PTPN6 gene on the malignant biological behavior of colon cancer cells. CO-IP assay was used to detect the interaction protein of PTPN6. PTPN6 was highly expressed in colorectal cancer tissues. High expression of PTPN6 is associated with poor prognosis in patients with colon cancer. PtPN6 knockdown inhibited the proliferation, invasion, migration, and clonogenesis of colorectal cancer LOVO and SW480 cells. At the same time, the knockdown of PTPN6 inhibited the EMT process in colorectal cancer. CO-IP results showed that PTPN6 had a protein-protein interaction with EGFR. Overexpression of EGFR increased the carcinogenic effect of PTPN6. The high expression of the PTPN6 gene can promote the proliferation, migration, and invasion of colon cancer cells. PTPN6 can interact with EGFR. PTPN6-EGFR complex may be an important factor affecting the biological characteristics of colon cancer cells and a potential therapeutic target.

## 1. Introduction

Colon cancer is the third most common malignancy in the world and the fourth most deadly [1–3]. Colon cancer poses a serious threat to human health [4]. Metastases are the leading cause of death in patients with colon cancer [5]. Further study on the mechanism of colon cancer metastasis is of great significance to improve the prognosis of patients [6, 7].

EMT process is an important biological process for epithelial cells of malignant tumors to acquire the ability of migration and invasion [8–12]. Therefore, the research on the molecular mechanism of EMT in malignant tumor cells, the exploration of new diagnostic methods based on the key regulatory molecules of EMT and the boot treatment methods is currently the focus of tumor metastasis research [13–15]. Protein-protein interactions are usually those in which two or more proteins interact to perform biochemical functions [16]. Proteins are the executors of life. However, the functions and behaviors in the life activity signal network are not completed by a single protein, which often needs the interaction between protein and nucleic acid and between protein and protein. Protein interaction networks are of great significance for the study of various signal networks and transcriptional regulation processes in living organisms [17].

PTPN6, a nonreceptor protein tyrosine phosphatase 6, is considered to be a major regulator of inflammation [18]. Studies have shown that PTPN6 can inhibit caspase-8 and Ripk3/Mlkl-dependent inflammation [19]. PTPN6 inhibits inflammation. PTPN6 restricts IL-1*α*-induced neutrophilic dermatosis by blocking syphilis kinase activation [20]. Furthermore, inhibition of PTPN6 aggravates alumina nanoparticle-induced COPD in mice by activating the STAT pathway [21]. The epidermal growth factor receptor (EGFR) signaling pathway is usually closed in normal tissues but activated and opened in tumor tissues. Moreover, EGFR activity was positively correlated with tumor progression. These results indicate that the EGFR signaling pathway is involved in the regulation of tumor development.

In this study, we detected the expression of PTPN6 in different colon cancer cell lines. PTPN6 overexpression vector and siRNA knockdown were constructed and transiently transfected into human colon cancer cells, respectively, to investigate the effect of overexpression of the PTPN6 gene on proliferation, migration, and invasion of colon cancer cells in vitro. Meanwhile, the interaction protein of PTPN6 was studied. The purpose of this study is to provide new ideas for the treatment of colon cancer.

## 2. Methods

### 2.1. Colon Cancer Tissue and Clinical Information Collection

Twenty cases of CRC patients admitted to the colorectal surgery department of our hospital from January 2020 to March 2021 were selected. There were 17 males and 7 females. The median age was 58 years (range: 42 to 71 years). All CRC patients were diagnosed by histopathology. All patients had not received radiotherapy, chemotherapy, integrated traditional Chinese and western medicine treatment, or immunotherapy before surgery. No other tumors, autoimmune diseases, and infectious diseases were found. CRC tissue and corresponding adjacent normal colorectal mucosal tissue (confirmed by histopathological section) ≥3 cm from the tumor were surgically removed as specimens. Immediately after being isolated, they were stored in liquid nitrogen. This study has been approved by the ethics committee of the General Hospital of Chinese PLA. All patients gave informed consent and signed informed consent.

### 2.2. Cell Culture

Cells were cultured in a DMEM cell medium containing 10% fetal bovine serum and cultured at 37°C in a 5% CO_2_ incubator. When the number of cells reached 60%–70% fusion, the cells were digested with 0.25% trypsin. The subculture was performed at a ratio of 1 : 3. The number of cells was adjusted to 1 × 10^8^/ml. After counting, they were inoculated in the corresponding culture plates for subsequent experiments.

### 2.3. Cell Transfection

The logarithmic growth phase cells were digested with trypsin and seeded into 6-well plates. When the confluence degree of cells in the plate reached 80%, plasmid pcDNA3.1-PTPN6/EGFR and plasmid pcDNA3.1 were transfected according to the instructions of the Lipofectanine™2000 transfection reagent. The ratio of the plasmid to Lipofectanine™ 2000 was 100 : 150. Only plasmid was added in one well of 6-well plate as control. One well was conventionally cultured cells. Moreover, 24 h after transfection, mRNA was detected by qPCR to identify the transfection efficiency.

### 2.4. QRT-PCR

Total RNA was extracted from cells according to the instructions of the Trizol reagent. The extracted total RNA will be used as a template and reverse-transcribed into cDNA for storage. Add samples according to the instructions of the qPCR kit. Take GAPDH as an internal reference. The sequence of primer was as follows: PTPN6, F: 5′-GGCCTGGACTGTGACATTGA-3′, R: 5′-ATGTTCCCGTACTCCGACTC-3′; GAPDH, F: 5′-AGGTGAAGTCGGAGTCAACG-3′, R: 5′-AGGGGTCATTGATGGCAACA-3′. Reaction conditions were as follows: predenaturation at 94°C for 30 s, 94°C for 10 s, 60°C for 30 s; 40 cycles were amplified, dissolution curves were detected, and three duplicate holes were set for each sample. The relative gene expression was calculated by 2^−ΔΔCt^. The experiment was repeated for 3 times.

### 2.5. Scratch Test

The cell density was adjusted to 2 × 10^4^/ml in the medium containing 10% fetal bovine serum. According to the 2 ml system, the cells were inoculated in 6-well plates with uniform horizontal lines on the back and cultured for 24 h. When the confluence of cells reaches over 90%, draw a flat line with the tip of 200 *μ*l sampling gun perpendicular to the horizontal line. Rinse with PBS twice to remove scratched cells. The photo position was marked and the track width was measured under an inverted microscope. The culture was continued at 37°C and 5% CO_2_. The distance between the scratches was observed and the percentage of cell migration was calculated under an inverted microscope at 0 and 24 hours after the scratches. Cell migration rate (%) = (measured spacing − scratch spacing)/measured spacing × 100%.

### 2.6. CCK-8 Was Used to Detect Cell Proliferation

The cells of the experimental group, control group, and conventional cell culture control group were cultured for 24 h, digested with trypsin, and suspended in a medium containing 10% fetal bovine serum. The cells were seeded into 96-well plates with 6 wells in each group, 1000 cells in 100 *μ*l medium per well. CCK-8 reagent was added to each well 72 hours after cell attachment. After incubation in the incubator for 4–6 h, the optical density (*D*) at 450 nm was determined by a microplate analyzer. The cell growth curve was drawn according to the detection value, and the cell proliferation was analyzed. The experiment was repeated for 3 times.

### 2.7. Transwell Experiment

Serum-free medium was used for starvation culture for 4 h. Trypsin was digested and suspended in a serum-free medium. The cell density was adjusted and cell suspension (containing 1 × 10^5^ cells) was added to each cell. The upper part of the compartment was 20 *μ*l Matrigel glue, and the lower part was added with 600 *μ*l complete medium. After conventional culture for 24 h, the chamber was removed and gently wiped with the cotton swab. The chamber was fixed in 4% paraformaldehyde for 10 min. Wash three times with PBS and let dry at room temperature. Stain with crystal violet solution for 20 min, wash PBS, and let dry at room temperature. The cells in 10 fields were counted under the microscope, and the average value was calculated to compare the differences between the experimental group and the control group.

### 2.8. Clone Formation Experiment

The cells of the experimental group, control group, and conventional cell culture control group were cultured for 24 h. The cell density was digested with trypsin and adjusted to 2 × 10^4^/ml in a medium containing 10% fetal bovine serum. In total, 5000 cells were inoculated in each well in a 6-well plate and routinely cultured for 1 week. After washing with PBS for 2 times, 4% paraformaldehyde was fixed for 15 min. Rinse on PBS and let dry at room temperature. Then, stain with crystal violet solution for 15 min, wash with PBS 3 times, and dry at room temperature. Under the inverted microscope, the number of clones was counted as one clone if the number of cells was greater than 50.

### 2.9. Immunocoprecipitation (CO-IP)

Use colon cancer cells and discard the culture medium. The cells were washed three times with 1 × PBS precooling. A specific cell lysate for immunoprecipitation was added and a certain proportion of protease inhibitors (such as PMSF with a final concentration of 1 mM) was mixed. Mix well and place in ice ultrasonic cell crushing machine to process until protein is extracted. In a precooled high-speed refrigerated centrifuge, centrifuge at 4°C, 14000 rpm, 10 min, discard precipitation. Collect the supernatant and store it on ice for later use or in a −80°C refrigerator. Magnetic bead pretreatment was processed. The antibody was diluted with binding buffer to prepare the antibody working solution with a final concentration of 25 ug/mL. Add 200 ul antibody working solution and mix with immunomagnetic beads. After 15 min, it was placed on a magnetic shelf for magnetic separation. Add 200 ul of binding buffer to 1.5 ml EP tubes. The bead-antibody complex is uniformly dispersed by blowing and then slowly approaches the magnetic rack for magnetic separation. Slightly blow with a pipette to evenly disperse the antigen and the bead-antibody complex. The reaction was carried out overnight in a 4°C overturn mixer. The magnetic bead-antibody-antigen complex, which had completed antigen adsorption, was magnetically separated. Wash EP tube with 200 ul detergent buffer and gently blow with a pipettor to evenly disperse the magnetic bead-antibody-antigen complex. Magnetic separation was then performed and the supernatant was discarded. Remove the EP tube from the magnetic rack; add 25 ul 1 × SDS-PAGE loading buffer into it and mix well and heat at 95°C for 5 min. Magnetic separation is then performed. The supernatant was collected for the SDS-PAGE test.

### 2.10. Western Blot

The cells to be measured were inoculated at a density of 1 × 10^5^ cells/well and cultured in 6-well plates for 24 h. The supernatant was absorbed and transfected for another 24 h. Cells in each group were collected and lysed with cell lysate. The supernatant was collected after centrifugation at 12000*g* 4°C for 15 min. The concentration of extracted protein was determined by BCA kit. Moreover, 50 *μ*g of the protein was isolated by SDS-PAGE. The isolated protein was electrically transferred to the PVDF membrane. Add 3 ml 5% skimmed milk powder sealant to the antibody incubator box. The membrane was sealed on a shaker at room temperature for 1 h and washed with TBST solution for 3 times, 10 min each time. Primary antibody (1 : 1000) was added and incubated overnight at 4°C. The membrane was cleaned with TBST solution for 3 times, 10 min each. HRP-labeled secondary antibody (1 : 2000) was added and incubated at room temperature for 1 h. The membrane with TBST was washed for 3 times, 10 min each. Finally, an appropriate amount of luminescent solution (liquids A and B were mixed in equal volume) was taken and the membrane was incubated for 3 min. In the gel imaging system, the protein content was analyzed by exposure and Quantity-One software, and GAPDH was used as internal reference.

### 2.11. Immunofluorescence

Dust up the culture medium in the Petri dish. The cells were washed twice with PBS at room temperature. Then, 4% paraformaldehyde solution was slowly dropping and fixed at room temperature for 20 min. After perforating, cells were sealed with 3%BSA for 1 h and washed with cold PBST for 3 times, 5 min/time. Add primary antibody and incubate overnight at 4°C. The Petri dish oscillates on a low-temperature shaker. Clean with cold PBS for 3 times, 5 min/time. Fluorescent-labeled secondary antibodies were incubated at room temperature for 2 h. Cold PBS cleaning was performed twice, 5 min/time. Cover glass was used and store at 4°C away from light. Fluorescence microscopic observation was performed.

### 2.12. Xenograft Model

Nude mice (4 weeks old, 18–20 g) were purchased from Beijing Vital River Laboratory Animal Technology Co., Ltd. After 1 week of adaptive feeding, the implant site was selected in the middle and posterior axillary region with a rich blood supply. Colon cancer cells with a logarithmic growth phase of 85% density were selected. After trypsin digestion, the serum in the cells was removed by washing twice with precooled PBS. Cells were beaten and counted in a serum-free medium, and the concentration of cells was adjusted to about 5 × 10^7^ cells/ml. Cells were inoculated subcutaneously into nude mice within half an hour after digestion. Wipe the needle area with an alcohol cotton ball. The injection volume was 150 ul/only. Use a cotton swab to compress the needle for 30 seconds to reduce the outflow of cell suspension from the needle. Five days after inoculation, a subcutaneous mass may begin to appear. Tumor growth in each group was closely monitored. Use a vernier caliper daily to measure the longest and shortest sites of the tumor. Tumor volume was counted using *V* = 1/2 × *A* × *B*^2^ (*A* is the long axis; *B* is the short axis). After 28 days, the nude mice were killed by cervical dislocation, and the tumor was removed and photographed. They were stored in liquid nitrogen for subsequent experiments and analysis. This study was approved by the ethics committee of the General Hospital of Chinese PLA.

### 2.13. Statistical Analysis

SPSS 22.0 statistical software was used for data analysis. Measurement data were expressed as mean ± standard deviation. The mean between the two groups was compared using the independent sample *t*-test. The mean between the groups was compared by a one-way analysis of variance. *P* < 0.05 indicated a statistically significant difference.

## 3. Results

### 3.1. Increased Expression of PTPN6 in Colon Cancer Tissues and Cell Lines

**Figure 1:**
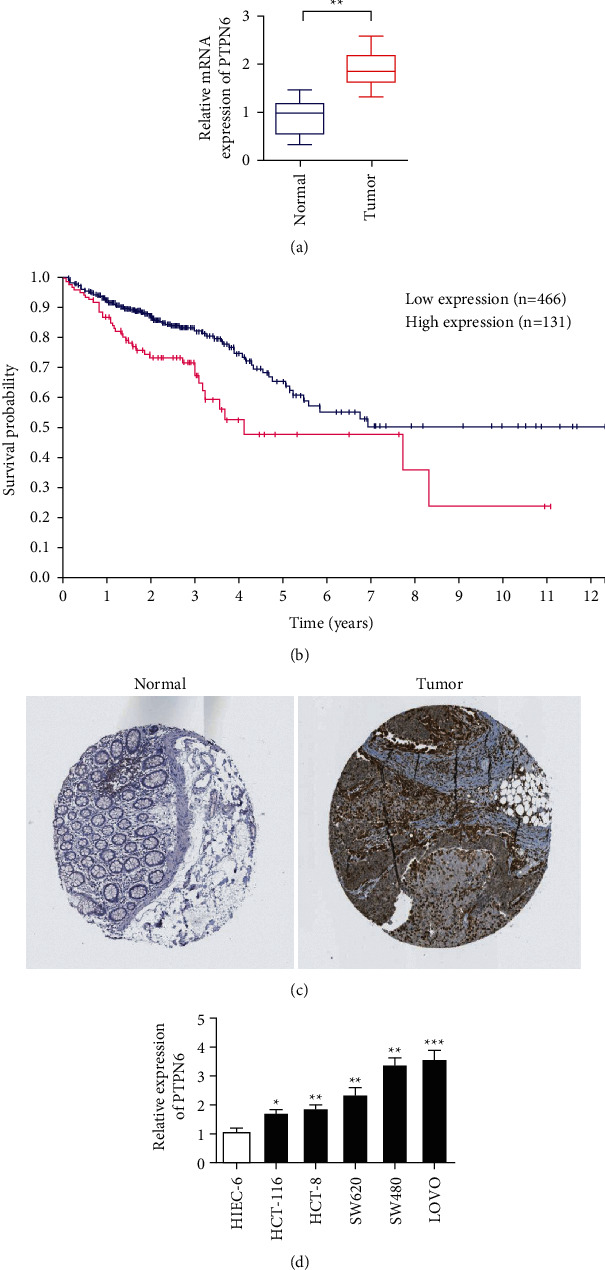
PTPN6 expression in colorectal cancer and adjacent tissues and analysis of clinical information with patients. (a) qRT-PCR detects the level of PTPN6 in adjacent tissues and colorectal cancer tissues. (b) Survival analysis of PTPN6 in colorectal cancer. Patients with high expression of PTPN6 have a poor prognosis. (c) Immunohistochemical detection of PTPN6 expression in adjacent tissues and colorectal cancer tissues. (d) The expression level of PTPN6 in normal intestinal epithelial cells and colorectal cancer cells. ^*∗*^*P* < 0.05, ^*∗∗*^*P* < 0.01,  and  ^*∗∗∗*^*P* < 0.001.

The qPCR results showed that PTPN6 mRNA was expressed in colon cancer tissues in adjacent normal tissues (Figure 1(a)). Results of survival analysis showed that patients with high expression of PTPN6 had a short survival time. The expression of PTPN6 was associated with poor prognosis (Figure 1(b)). Immunohistochemical results showed that the expression of PTPN6 protein in colon cancer tissue was consistent with the expression trend of PTPN6 mRNA. The expression of PTPN6 protein was significantly increased in colon cancer tissues (Figure 1(c)). The results of qPCR method showed that the relative expression levels of PTPN6 mRNA in HCT-116, HCT-8, SW620, SW480, LOVO, and other colon cancer cells were significantly higher than those in the control group (HIEC-6) (Figure 1(d)). Moreover, the expression in SW480 and LOVO cells was higher than that in the other three cells. SW480 and LOVO cell lines were selected for subsequent PTPN6 gene experiments.

### 3.2. Knocking Down PTPN6 Inhibited the Proliferation, Invasion, Migration, and Clone Formation of Colorectal Cancer LOVO and SW480 Cells

**Figure 2:**
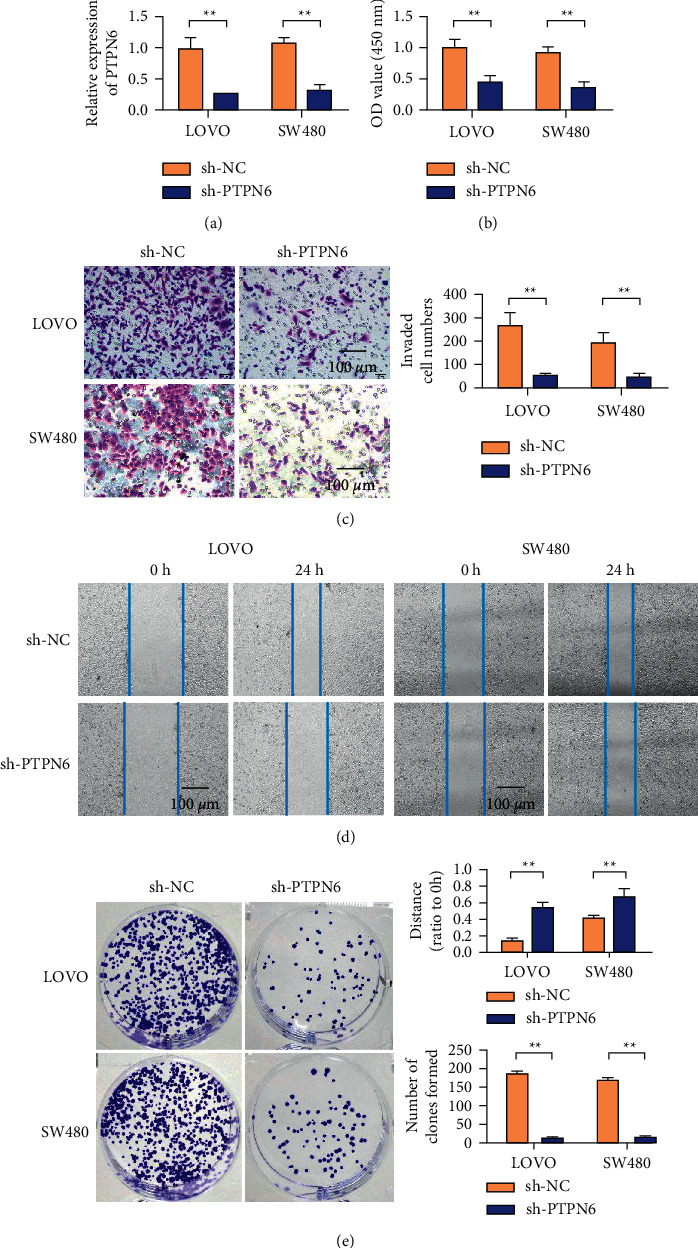
Knockdown of PTPN6 inhibits the proliferation, invasion, migration, and clonogenesis of colorectal cancer LOVO and SW480 cells. (a) PTPN6 expression efficiency detection in LOVO and SW480 cells. (b) LOVO and SW480 cell proliferation detection. (c) Transwell detection of LOVO and SW480 cells. (d) LOVO and SW480 cell scratched detection. (e) Detection of cloning ability of LOVO and SW480 cells. ^*∗∗*^*P* < 0.01, magnification 400x.

The qPCR results showed that the relative expression of PTPN6 mRNA in sh-PTPN6 transfected cells was significantly lower than that in sh-NC cells (Figure 2(a)). The results of the CCK-8 experiment (Figure 2(b)) showed that the proliferation ability of SW480 and LOVO cells knocked down by the PTPN6 gene was significantly lower than that of the control group, with statistical significance. Transwell compartment invasion assay (Figure 2(c)) showed that the number of cells passing through the artificial basal membrane in SW480 and LOVO cells with knockdown of PTPN6 gene was significantly lower than that in the control group transfected with empty body cells. Scratch healing experimental results (Figure 2(d)) showed that the scratch healing rate of SW480 and LOVO cells knocked down by the PTPN6 gene was significantly lower than that of the empty vector transfected control group. The results of the clone formation experiment (Figure 2(e)) showed that the clone number of SW480 and LOVO cells knocked down by PTPN6 gene was significantly lower than that of the empty vector transfected control group.

### 3.3. Knocking Down PTPN6 Inhibited Colon Cancer EMT in SW480 and LOVO Cells

**Figure 3:**
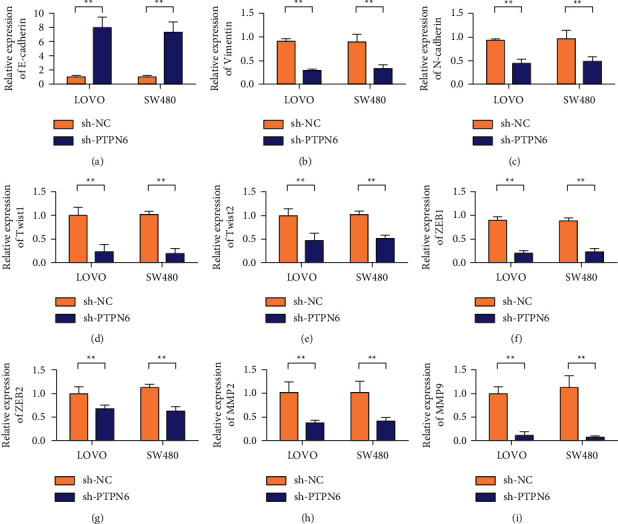
Knockdown of PTPN6 inhibits colon cancer EMT in SW480 and LOVO cells. (a) In SW480 and LOVO cells, the expression of E-cadherin when PTPN6 is knocked down was detected by qRT-PCR. (b) In SW480 and LOVO cells, the expression of Vimentin when PTPN6 is knocked down was detected by qRT-PCR. (c) In SW480 and LOVO cells, the expression of N-cadherin when PTPN6 is knocked down was detected by qRT-PCR. (d) In SW480 and LOVO cells, the expression of Twsit1 when PTPN6 is knocked down was detected by qRT-PCR. (e) In SW480 and LOVO cells, the expression of Twist2 when PTPN6 is knocked down was detected by qRT-PCR. (f) In SW480 and LOVO cells, the expression of ZEB1 was detected by qRT-PCR when PTPN6 was knocked down. (g) In SW480 and LOVO cells, the expression of ZEB2 when PTPN6 is knocked down was detected by qRT-PCR. (h) In SW480 and LOVO cells, the expression of MMP2 when PTPN6 is knocked down was detected by qRT-PCR. (i) In SW480 and LOVO cells, the expression of MMP9 when PTPN6 is knocked down was detected by qRT-PCR. ^*∗∗*^*P* < 0.01.

Further analysis showed that sh-PTPN6 significantly promoted the expression of the epithelial marker E-cadherin. The experimental results are shown in Figure 3(a). In addition, we found that PTPN6 knockdown inhibited the expression of mesenchymal markers Vimentin and N-cadherin (Figures 3(b) and 3(c)). Detection results of the expression levels of transcription factors with Twist1, TWSIT2, ZEB1, and ZEB2 showed that knockdown of PTPN6 could inhibit the expression of the above transcription factors, with statistical significance (Figures 3(d)–3(g)). The effects of shPTPEN6 on MMP2 and MMP9 were detected by qRT-PCR. The results showed that the expression levels of MMP2 and MMP9 in LOVO and SW480 cells decreased after PTPEN6 silencing (Figures 3(h) and 3(i)).

### 3.4. The Effect of sh-PTPN6 on Tumorigenic Ability of Colon Cancer Cells Was Detected by the Tumor Formation Test

**Figure 4:**
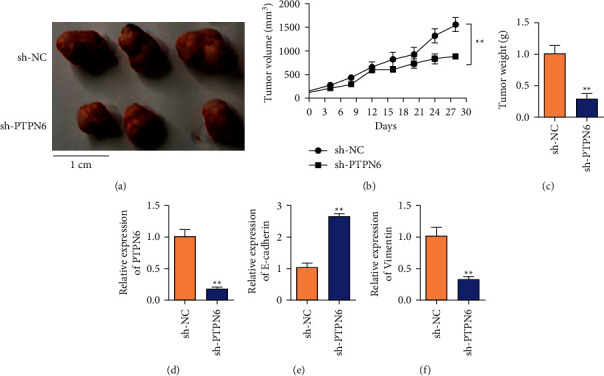
Knockdown of the PTPN6 gene inhibits the growth of colon cancer tumors. (a) Representative pictures of tumors in the control group and PTPN6 knockdown group. (b) Detection of the tumor volume of LOVO cells transfected with sh-PTPN6. (c) Detection of the tumor weight of LOVO cells transfected with sh-PTPN6. (d) Detection of the expression of PTPN6 in tumor tissues after sh-PTPN6 transfected LOVO cells are tumor-bearing. (e) Detection of the expression of E-cadherin in tumor tissues after sh-PTPN6 transfected LOVO cells are tumor-bearing. (f) Detecting the expression of Vimentin in tumor tissues after sh-PTPN6 transfected LOVO cells are tumor-bearing. ^*∗∗*^*P* < 0.01.

In order to further detect the effect of sh-PTPN6 on the tumor-forming ability of SW480 cells, the xenograft tumor model of nude mice was established by injecting SW480 colon cancer cells subcutaneously. Then, sh-NC and sh-PTPN6 treated colon cancer cells were implanted subcutaneously into the left anterior axilla of nude mice. Tumor volume was measured every day and tumor growth curves were plotted to compare the differences between the groups. Tumor volume was measured at 28 days after death, and the differences in tumor volume and weight were compared. The results showed that the volume and size of the transplanted tumor in the sh-PTPN6 group were significantly lower than those in the control group (Figures 4(a) and 4(b)). *P* < 0.01). The weight of transplanted tumors in the sh-PTPN6 group was also lower than that in the sh-SN group, and the difference was statistically significant (*P* < 0.01, Figure 4(c)). The expression of PTPN6 in the sh-NC and sh-PTPN6 group was detected by qRT-PCR. The results showed that the expression of PTPN6 was decreased in sh-PTPN6 (Figure 4(d)). However, after the knockdown of PTPN6, the expression of E-cadherin increased (Figure 4(e)) and the expression of Vimentin decreased (Figure 4(f)).

### 3.5. PTPN6 Has Protein-Protein Interaction with EGFR

**Figure 5:**
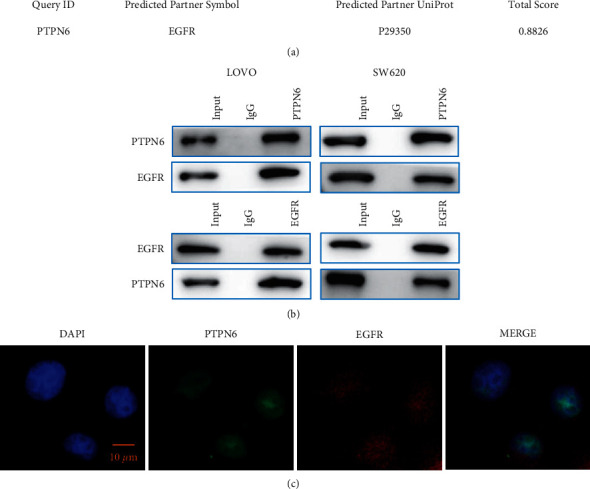
Protein-protein interaction between PTPN6 and EGFR. (a) PTPN6 interaction protein prediction. (b) CO-IP experiment verifies the interaction between PTPN6 and EGFR. (c). Immunofluorescence colocalization of PTPN6 and EGFR in colon cancer cells. ^*∗∗*^*P* < 0.01.

We used bioinformatics methods to predict PTPN6 interacting proteins (FpClass: https://dcv.uhnres.utoronto.ca/FPCLASS/ppis/). The results showed that EGFR may be the interacting protein of PTPN6 (Figure 5(a)). CO-IP experiment further verified the interaction between PTPN6 and EGFR (Figure 5(b)). PTPN6 specific antibody was used as the primary antibody and corresponding secondary antibody for development detection. As shown in the results, EGFR protein could be detected in both the CO-IP product and the cell total protein lysate sample, while no corresponding bands were detected in the negative control. Similarly, CO-IP of PTPN6 bands can be produced with EGFR primary antibody. In space, in order to further clarify the interaction between PTPN6 and EGFR, we performed immunofluorescence detection of PTPN6 and EGFR in colon cancer cells. The nuclei were stained and mapped using blue fluorescent-labeled DAPI. Immunofluorescence localization showed that PTPN6 could interact with EGFR in space. Both of them are located in the cytoplasm, and there is colocalization (Figure 5(c)).

### 3.6. Overexpression of EGFR Increased the Cancer-Promoting Effect of PTPN6

**Figure 6:**
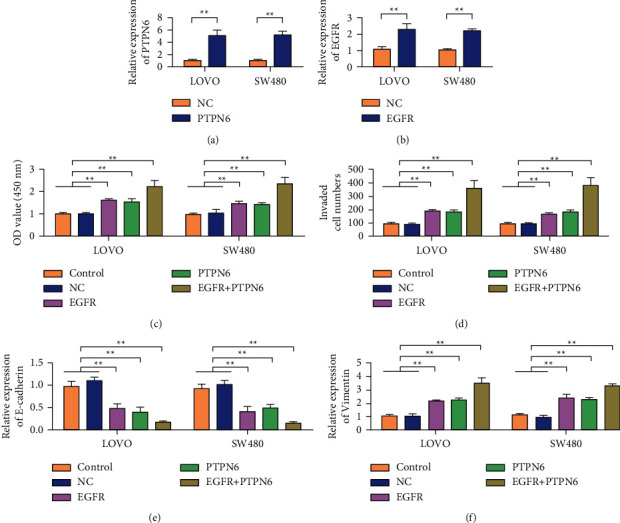
Overexpression of EGFR increases the cancer-promoting effect of PTPN6. (a) Detection of PTEN6 expression in LOVO and SW480 cells. (b) Detection of EGFR expression in LOVO and SW480 cells. (c) Detection of the cell proliferation rate of LOVO and SW480. (d) Transwell detection of LOVO and SW480 cells. (e) Detection of E-cadherin expression in LOVO and SW480 cells. (f) Detection of Vimentin expression in LOVO and SW480 cells. ^*∗∗*^*P* < 0.01.

Detection results of PTEN6 expression in LOVO and SW480 cells showed that compared with the control group, the expression of PTPN6 was increased in the PTPN6 plasmid transfection group (Figure 6(a)). Detection of EGFR expression in LOVO and SW480 cells showed that EGFR expression was increased in the EGFR plasmid transfected group compared with the control group (Figure 6(b)). Cell proliferation assay showed that both PTPN6 and EGFR overexpression could promote cell proliferation. However, cell proliferation was strongest in the group with both PTPN6 and EGFR overexpression (Figure 6(c)). Transwell assay results showed that both PTPN6 and EGFR overexpression promoted cell invasion ability. However, the cells in the group with both PTPN6 and EGFR overexpression showed the strongest invasion ability (Figure 6(d)). Simultaneously, overexpression of PTPN6 and EGFR inhibited the expression of E-cadherin (Figure 6(e)). However, it can upregulate the expression of Vimentin (Figure 6(f)).

## 4. Discussion

The invasion and metastasis of malignant tumors is a complex process of multigene involvement and multistep regulation [22–25]. The EMT process of tumor cells directly causes the weakening of the adhesion between tumor cells in situ and the radical change of cell polarity [26]. The ability of cells to migrate during EMT is enhanced, which is an early event of tumor metastasis. The regulation of gene expression plays a key role in the EMT process of tumor cells [27]. Therefore, studying the molecular mechanism of EMT-related genes regulated by specific factors in colon cancer cells is of great value for understanding the mechanism of colon cancer metastasis and identifying inhibitory targets [28, 29].

As a cytoplasmic phosphatase, PTPN6 is mainly expressed in hematopoietic cells and epithelial cells. PTPN6 is a negative regulator of multiple signaling pathways [30, 31]. PTPN6 plays different roles and mechanisms in regulating cell cycle and cell proliferation in different types of tumors. Plutzky et al. [32] first observed the presence of PTPN6 in frequently damaged chromosomal regions in childhood leukemia. Oka et al. [33] observed downregulation of mRNA and protein expression of PTPN6 gene in natural killer T cell lymphoma and 95% of several other types of malignant lymphoma. Thus, loss of PTPN6 expression may lead to malignant transformation and increased aggressiveness. PTPN6 gene expression is downregulated in advanced chronic myelogenous leukemia, breast cancer, and liver cancer. Moreover, the expression level of PTPN6 is related to malignant phenotypes such as malignant degree, invasion, and metastasis of tumors and plays a role of tumor suppressor genes in the development of tumors [34, 35].

In this study, the expression of PTPN6 gene in colon cancer cell lines was firstly confirmed by experiments. The results of CCK-8 and clone formation assay showed that the proliferation ability of colon cancer cells transfected with PTPN6 overexpressing plasmid was significantly enhanced in vitro. The results of scratch healing and transwell invasion assay showed that in vitro migration and invasion ability of colon cancer cells were significantly increased after transfection of PTPN6 overexpressing plasmid. These results suggest that the PTPN6 gene may promote the proliferation and invasion ability of colon cancer cells in vitro. This is consistent with clinical data analysis. Clinical data analysis also showed that PTPN6 was highly expressed in colon cancer tissues. Patients with high expression of PTPN6 have a poor prognosis.

Proteins do not usually function as a single substance but as members of a dynamic network that acts in concert to allow cells to function. There is growing evidence that protein-protein interactions are critical in the biological processes of living cells. Finally, in order to further study the role of PTPN6 in the cell signaling network, we predicted and verified that EGFR could interact with PTPN6 according to experiments. EGFR is a membrane receptor with tyrosine protein kinase (TK) activity. EGFR can be activated by epidermoid growth factor and transmit information to the nucleus along multiple downstream signaling pathways, acting on target genes and participating in the regulation of the occurrence and development of a variety of tumors. Ciardiello and Tortora [36] reported that after activation of EGFR by growth factors, cell proliferation, adhesion, movement, and proteolysase secretion could be regulated through PI3K/Akt or MAPK downstream signaling pathways.

## 5. Conclusion

In conclusion, this study suggests that PTPN6 gene may be an oncopromoter gene in colon cancer. It also plays an important role in regulating colon cancer cell proliferation, migration and invasion. Mechanism studies showed that PTPN6 can interact with EGFR, which is expected to be a molecular target for the treatment of colon cancer.

## Data Availability

The data used to support the findings of this study are available from the corresponding author upon request.

## References

[1] Garrett W. S. (2019). The gut microbiota and colon cancer. *Science*.

[2] Zhou B., Zong S., Zhong W. (2019). Interaction between laminin-5*γ*2 and integrin beta1 promotes the tumor budding of colorectal cancer via the activation of yes-associated proteins. *Oncogene*.

[3] Lannagan T. R., Jackstadt R., Leedham S. J., Sansom O. J. (2021). Advances in colon cancer research: in vitro and animal models. *Current Opinion in Genetics & Development*.

[4] Grothey A., Sobrero A. F., Shields A. F. (2018). Duration of adjuvant chemotherapy for stage III colon cancer. *New England Journal of Medicine*.

[5] Tauriello D. V. F., Palomo-Ponce S., Stork D. (2018). TGF*β* drives immune evasion in genetically reconstituted colon cancer metastasis. *Nature*.

[6] Arlt F., Stein U. (2009). Colon cancer metastasis: MACC1 and Met as metastatic pacemakers. *The International Journal of Biochemistry & Cell Biology*.

[7] Zhong W., Hou H., Liu T. (2020). Cartilage oligomeric matrix protein promotes epithelial-mesenchymal transition by interacting with transgelin in colorectal cancer. *Theranostics*.

[8] Bates R. C., Mercurio A. (2005). The epithelial-mesenchymal tansition (EMT) and colorectal cancer progression. *Cancer Biology & Therapy*.

[9] Xi X., Chu Y., Liu N. (2019). Joint bioinformatics analysis of underlying potential functions of hsa-let-7b-5p and core genes in human glioma. *Journal of Translational Medicine*.

[10] Lv L., Yi Q., Yan Y., Chao F., Li M. (2021). SPNS2 downregulation induces EMT and promotes colorectal cancer metastasis via activating AKT signaling pathway. *Frontiers in Oncology*.

[11] Babaei G., Aziz S. G.-G., Jaghi N. Z. Z. (2021). EMT, cancer stem cells and autophagy; the three main axes of metastasis. *Biomedicine & Pharmacotherapy*.

[12] Nowak E., Bednarek I. (2021). Aspects of the epigenetic regulation of EMT related to cancer metastasis. *Cells*.

[13] Brabletz T., Kalluri R., Nieto M. A., Weinberg R. A. (2018). EMT in cancer. *Nature Reviews Cancer*.

[14] Singh A., Settleman J. (2010). EMT, cancer stem cells and drug resistance: an emerging axis of evil in the war on cancer. *Oncogene*.

[15] Meng J., Ai X., Lei Y. (2019). USP5 promotes epithelial-mesenchymal transition by stabilizing SLUG in hepatocellular carcinoma. *Theranostics*.

[16] Hu X., Harvey S. E., Zheng R. (2020). The RNA-binding protein AKAP8 suppresses tumor metastasis by antagonizing EMT-associated alternative splicing. *Nature Communications*.

[17] Singh M., Yelle N., Venugopal C., Singh S. K. (2018). EMT: mechanisms and therapeutic implications. *Pharmacology & Therapeutics*.

[18] Adhikari A., Martel C., Marette A., Olivier M. (2017). Hepatocyte SHP-1 is a critical modulator of inflammation during endotoxemia. *Scientific Reports*.

[19] Jia S. H., Parodo J., Charbonney E. (2014). Activated neutrophils induce epithelial cell apoptosis through oxidant-dependent tyrosine dephosphorylation of caspase-8. *American Journal Of Pathology*.

[20] Lukens J. R., Vogel P., Johnson G. R. (2013). RIP1-driven autoinflammation targets IL-1*α* independently of inflammasomes and RIP3. *Nature*.

[21] Li X., Yang H., Wu S. (2017). Suppression of PTPN6 exacerbates aluminum oxide nanoparticle-induced COPD-like lesions in mice through activation of STAT pathway. *Particle and Fibre Toxicology*.

[22] Prabhu A., Obi K. O., Rubenstein J. H. (2014). The synergistic effects of alcohol and tobacco consumption on the risk of esophageal squamous cell carcinoma: a meta-analysis. *American Journal of Gastroenterology*.

[23] Zhong W., Yang W., Qin Y. (2019). 6-Gingerol stabilized the p-VEGFR2/VE-cadherin/*β*-catenin/actin complex promotes microvessel normalization and suppresses tumor progression. *Journal of Experimental & Clinical Cancer Research*.

[24] Richard V., Kumar T. R. S., Pillai R. M. (2021). Transitional dynamics of cancer stem cells in invasion and metastasis. *Translational Oncology*.

[25] Zhang X., Xue J., Yang H., Zhou T., Zu G. (2021). TNFAIP6 promotes invasion and metastasis of gastric cancer and indicates poor prognosis of patients. *Tissue and Cell*.

[26] Yilmaz M., Christofori G. (2009). EMT, the cytoskeleton, and cancer cell invasion. *Cancer and Metastasis Reviews*.

[27] Derynck R., Weinberg R. A. (2019). EMT and cancer: more than meets the eye. *Developmental Cell*.

[28] Georgakopoulos-Soares I., Chartoumpekis D. V., Kyriazopoulou V., Zaravinos A. (2020). EMT factors and metabolic pathways in cancer. *Frontiers in oncology*.

[29] Xi X., Liu N., Wang Q. (2019). ACT001, a novel PAI-1 inhibitor, exerts synergistic effects in combination with cisplatin by inhibiting PI3K/AKT pathway in glioma. *Cell Death & Disease*.

[30] De La Iglesia N., Konopka G., Lim K.-L. (2008). Deregulation of a STAT3–interleukin 8 signaling pathway promotes human glioblastoma cell proliferation and invasiveness. *Journal of Neuroscience*.

[31] Jia W.-Q., Wang Z.-T., Zou M.-M. (2018). Verbascoside inhibits glioblastoma cell proliferation, migration and invasion while promoting apoptosis through upregulation of protein tyrosine phosphatase SHP-1 and inhibition of STAT3 phosphorylation. *Cellular Physiology and Biochemistry*.

[32] Plutzky J., Neel B. G., Rosenberg R. D. (1992). Chromosomal localization of an SH2-containing tyrosine phosphatase (PTPN6). *Genomics*.

[33] Oka T., Yoshino T., Hayashi K. (2001). Reduction of hematopoietic cell-specific tyrosine phosphatase SHP-1 gene expression in natural killer cell lymphoma and various types of lymphomas/leukemias: combination analysis with cDNA expression array and tissue microarray. *American Journal of Pathology*.

[34] Wen L.-Z., Ding K., Wang Z.-R. (2018). SHP-1 acts as a tumor suppressor in hepatocarcinogenesis and HCC progression. *Cancer Research*.

[35] Chen Z., Shojaee S., Buchner M. (2015). Signalling thresholds and negative B-cell selection in acute lymphoblastic leukaemia. *Nature*.

[36] Ciardiello F., Tortora G. (2001). A novel approach in the treatment of cancer: targeting the epidermal growth factor receptor. *Clinical Cancer Research: An Official Journal of the American Association for Cancer Research*.

